# Genetic structure and selective sweeps in Kirghiz sheep using SNP50K bead chip

**DOI:** 10.3389/fgene.2024.1432105

**Published:** 2024-08-21

**Authors:** Xiaopeng Li, Lijun Zhu, Cheng-Long Zhang, Xueyan Wang, Yanhao Li, Wen Zhou, Zhipeng Han, Ruizhi Yang, Yuwei Peng, Yahui Han, Lulu Zhang, Langman Zheng, Shudong Liu

**Affiliations:** ^1^ College of Animal Science and Technology, Tarim University, Xinjiang, China; ^2^ Key Laboratory of Tarim Animal Husbandry Science and Technology, Xinjiang Production and Construction Corps, Xinjiang, China

**Keywords:** Kirghiz sheep, genetic patterns, selection signature, GO enrichment analysis, selective sweeps

## Abstract

The objective of this study is to analyze environmental genetic selection signals in large-scale sheep populations with conflicting environmental adaptations, aiming to identify and isolate genes associated with environmental adaptations in sheep populations. Kirghiz sheep, which inhabit high-altitude environments year-round, demonstrate the ability to adapt to extreme conditions. In this study, 42 Kirghiz sheep, 24 Tien-Shan in Kyrgyzstan sheep, 189 Qira black sheep, and 160 Chinese Merino sheep were genotyped using Illumina Ovine SNP50K chip. Regions exhibiting a selection signal threshold of 5%, as well as PI analysis and haplotype statistical scanning gene data were annotated, and intersecting genes were identified as candidate genes. Through Fst and haplotype statistical analysis revealed the key gene *PDGFD* and its vicinity’s impact on fat deposition in sheep tails. Additionally, Fst and PI analysis uncovered genes related to high-altitude adaptation as well as those linked to animal growth and reproduction.Further GO and KEGG enrichment pathway analyses unveiled pathways associated with high-altitude adaptation such as negative regulation of peptidyl-tyrosine phosphorylation and xenobiotic metabolism processes.This investigation into the adaptability of Kirghiz sheep provides theoretical support and practical guidance for the conservation and genetic enhancement of Kirghiz sheep germplasm resources.

## 1 Introduction

The varying altitude gradient on Earth leads to complex and diverse climates, and China’s topography exhibits a terraced structure due to these differences in altitude (Wu et al., 2023). Atushi City in Kezhou, Xinjiang, located in the southwest of the Tarim Basin, lies at the southern foot of the Tianshan Mountains with altitudes ranging from about 1,500 to 2,000 m. This area is characterized by undulating hills and peaks, with the highest point reaching 7,719 m (Qi et al., 2021). Kirghiz sheep, native to high-altitude regions, exhibit strong environmental adaptability and stable growth traits. Predominantly found in Wuqia County, Kezilesu Autonomous Prefecture, in the southern Tianshan Mountains of Xinjiang, these sheep are known for their tolerance to rough feeding, disease resistance, strong stress resistance, and rapid weight gain. They have adapted well to the cold high-altitude environment of the Pamirs in southern Xinjiang. Animals in high-altitude regions undergo complex physiological and behavioral adjustments to adapt to their harsh environment (Guo et al., 2021). These animals need to cope with extreme climatic conditions such as cold, hypoxia, and intense ultraviolet radiation to ensure their survival and reproduction (Castiglione et al., 2017).Adaptations to high-altitude environments were analyzed by genome-wide scanning of natural selection traits in each species. Kirghiz sheep as one of Xinjiang’s meat sheep,In the 1970s,Kirghiz sheep were endangered until 1974 when China restored the Kirghiz Autonomous Prefecture sheep farm, allowing for the protection of this breed.Currently, there are approximately 640,000 Kirghiz sheep in the Kirghiz Autonomous Prefecture. Although breeding efforts for Kirghiz sheep have been ongoing, there have been relatively few studies on their genetic structure and genetic diversity, resulting in incomplete population genetic structures and significant degradation of breed characteristics. Through in-depth research and identification of the gene types adapted to the environment (Porcelli et al., 2015), new breeds that can adapt to extreme environment and have excellent production performance can be effectively screened and developed (Williams et al., 2007), and the genetic potential of individual animals can be more accurately assessed (Wearing and Scott, 2022).

Gene chip technology plays an important role in gene selection and mapping, genome breeding, breed identification,and parentage testing (Li Y. et al., 2014).The 50 K SNP chip for sheep can evenly cover the entire sheep genome and includes important trait loci.Wu (Han and Xiong, 2001) discovered candidate genes related to growth traits and high-altitude adaptability in Cashmere goats from two different regions using the Fst and PI methods.Wang (Guo et al., 2019)compared and analyzed Tibetan sheep, Altay sheep, Duolang sheep,Hu sheep,and Mongolian sheep using sheep genome re-sequencing and found genes related to hemoglobin levels and red blood cell counts in certain selection regions, such as the CYP17 gene on chromosome 22 and the DNAJB5 gene on chromosome 2 of Duolang sheep.Caiye Zhu (Wang et al., 2019)studied seven sheep breeds from different regions using the 50 K SNP chip technology and identified candidate genes associated with high-altitude adaptability through Fst and XP-EHH analysis, such as EPAS1,CRYAA, LONP1,NF1,DPP4,SOD1,PPARG, SOCS2 and detected 31 significant SNPs associated with tail type traits in indigenous Chinese sheep and identified BMP2 and PDGFD as candidate genes for tail type traits.Based on the identified candidate genes for tail type traits.Baazaouil (Caiye et al., 2023) conducted whole-genome sequencing of semi-arid sheep using this chip and identified candidate genes PROKR1 and BMP2 related to tail fat. Caiye Zhu (Baazaoui et al., 2021) use a total of 31 significant SNPs related to tail type traits were detected in Chinese native sheep, and BMP2 and PDGFD were identified as tail type trait candidate genes. Based on the above identified tail type trait candidate genes, BMP2 and PDGFD genes were selected to study the relationship between tail SNPs of Altay sheep and Tibetan sheep. (Wei et al., 2016) conducted genome selection on 140 individuals of indigenous Chinese sheep breeds and determined that PDGFD may affect fat deposition in fat-type sheep.

Population genetic diversity and selection signature are among the fundamental methods for studying sheep genomes and can reveal how genes respond to environmental selection. Therefore,the aim of this study was to use genome selection and haplotype statistics to study the population structure and genetic diversity of Kirghiz sheep, identify genetic markers associated with production traits,and provide theoretical basis for the conservation of sheep germplasm resources in southern Xinjiang,to promote animal husbandry sustainable development.

## 2 Materials and methods

### 2.1 Animal care

The study followed the guidelines of the Ethics Committee of Tarim University of Science and Technology (SYXK 2020-009) for animal experiments.

### 2.2 Experimental animals

In this study, 189 blood samples from adult Qira black sheep (QR) and 42 venous blood samples from adult Kirghiz sheep (KE) were randomly collected from Jinken Aoqun Agriculture and Animal Husbandry Technology Limited in Qira County, Hotan District and Bozheng Sheep Industry Technology Limited in Wuqia County, Kezhou. In addition, data from 160 Chinese merino sheep (CM) and 24 Tien-Shan in Kyrgyzstan (TNSH) were used.The data are from:https://doi.org/10.5061/dryad.37pvmcvff (Deniskova et al., 2019a).

### 2.3 DNA extraction and identification

The remaining samples of genomes DNA (gDNA) underwent agarose gel electrophoresis and nanodrop ND-2000 (Thermo Scientific) concentration analysis to ensure their quality and concentration.After adjusting the gDNA concentration to 50 ng/L,whole-genome amplification was performed. Subsequently,the gDNA was fragmented and precipitated before being re-suspended in hybridization buffer. The re-suspended DNA fragments were then applied to the chip for hybridization.Following hybridization,non-specifically bound DNA was removed, leaving behind specifically bound sites. These specific binding sites were then subjected to single-base extension and staining, followed by scanning using the Illumina iScan Reader. After completing the scan,the extracted DNA underwent quality control to obtain detailed quality control reports. Finally,the raw data scanned by the iScan system were imported into the Illumina official data analysis pipeline to generate PLINK files.

### 2.4 Genetic diversity and population structure

We utilized liftover (https://genome.ucsc.edu/cgi-bin/hgLiftOver) to convert the genome coordinates of 37 Tien-Shan sheep, then merged the files and performed quality control on the SNP data using PLINK software (version 1.90). We used the following criteria: inclusion detection rate less than 90% and hardy-Weinberg balance test *p*-value less than 10^–5^ (Zhang et al., 2022).

### 2.5 Genetic diversity and selection signature

The PLINK (V1.90) software was used to perform principal component analysis based on the variance-standardized relationship matrix of the quality-controlled data. The NJ matrix was calculated using VCF2Dis (https://github.com/BGI-shenzhen/VCF2Dis) to draw the evolutionary tree, and the sparse non-negative matrix factorization algorithm sNMF (http://membres-timc.imag) was employed to generate estimates of ancestral proportions.

### 2.6 Fixation index

Fst is a statistical test used to measure the degree of differentiation between populations, mainly used to study the extent of genetic variation between different populations,as well as population structure and genetic diversity.The calculation formula is as follows:

$$Fst=\frac{MSP-MSG}{MSP+\left(n_{c}-1\right)MSG}$$

In this formula,MSG rep resents the mean square error of the detected intra-group sites, while MSP stands for the mean square variance of the inter-group sites, indicating the corrected average sample size between groups (Zhang W. et al., 2023). In this study,SNP sites retained were calculated using the unbiased estimation Fst method, yielding comparisons between Kirghiz sheep and Qira black sheep, as well as Kirghiz sheep and Chinese Merino sheep.

### 2.7 Haplotype analysis

We conducted haplotype statistics and inference on the *PDGFD* gene region genotype data of Kirghiz sheep and Tien-Shan sheep using the genehap R package in R scripts (Zhang R. et al., 2023).With this software, we combined continuous SNP data into individual haplotypes and generated corresponding haplotype information for each individual. We employed the genehap R software for phenotype-associated haplotype statistics in the vicinity of the *PDGFD* gene region.

### 2.8 Nucleotide diversity (PI)

Nucleotide diversity (PI) refers to the average number of different nucleotide at the same position of a random sequence taken from the DNA of multiple samples in a population, representing the degree of nucleotide polymorphisms within the population (Brouillette et al., 2000). The calculation formula is as follows:

$$PI=\sum_{j=i}^{S}h_{j}$$

In the above formula, S represents the number of segregating sites, and $h_{j}$ represents the heterozygosity of the j segregating site. We utilized the vcftools software to compute population nucleotide diversity.

### 2.9 Enrichment analysis of candidate genes

We conducted statistical analysis of the *p*-values for Fst and PI calculated for the Kirghiz sheep (KE), Chinese Merino sheep (CM), and Qira black sheep (QR) populations,and plotted Manhattan distribution graphs. The top 5% of selected sites were referenced against two databases: the sheep genome Ovis Oar_v4.0 for annotation and the NCBI database (http://www.ncbi.nlm.nih.gov/gene) for enrichment analysis using cluster profile (Yu et al., 2012). The KEGG and GO enrichment analyses covered three aspects:Biological Process, Cellular Component, and Molecular Function.

## 3 Results

### 3.1 Population structure

**FIGURE 1:**
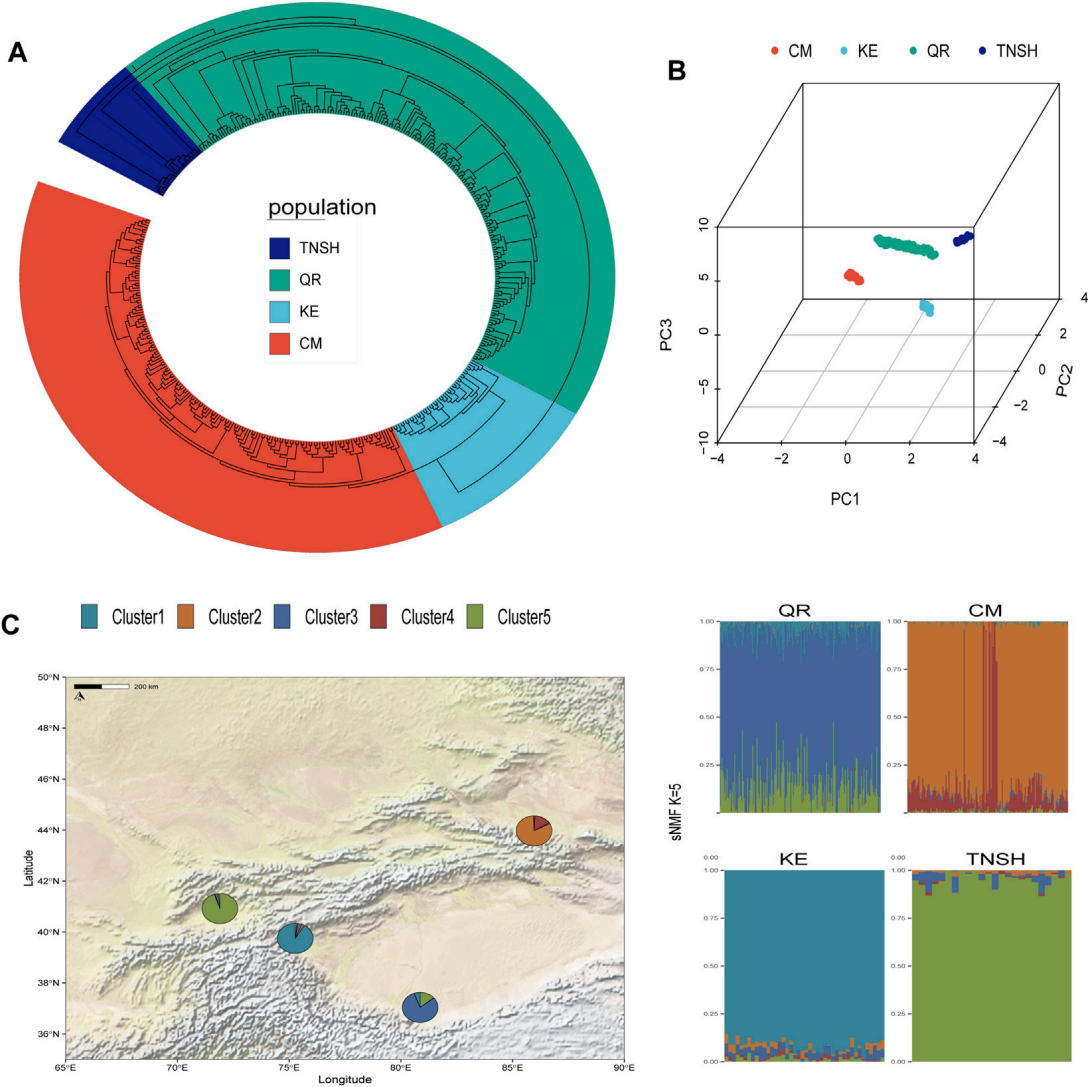
**(A)** NJ evolutionary tree. **(B)** Principal Component Analysis of Four Sheep Breeds (*X*-axis represents PC1, *Y*-axis represents PC2, *Z*-axis represents PC3. **(C)** sNMF Ancestral Proportion Estimation (minimized CV_error when K = 5).

Cluster analysis and principal component analysis (PCA) were conducted on SNP data from 415 sheep. The four populations were clustered into four groups, and significant differentiation among them was observed using PC1, PC2, and, PC3. TNSH showed slight differentiation compared to other populations, while QR exhibited closer proximity to TNSH in terms of principal components, consistent with the results of the evolutionary tree. The four sheep breeds QR, KE, CM, and TNSH formed separate branches, with KE being distantly separated from the other three sheep breeds.QR and TNSH populations showed closer structural proximity, with QR extending outward, indicating the separation of KE from the other three sheep breeds (Figure 1B). Considering the different evolutionary processes among the four sheep breeds, we further inferred the ancestral proportions of the four distinct sheep populations using the sNMF software (Figure 1C). When K = 5, each of the four sheep breeds exhibited distinct ancestral components, consistent with the separation observed in the PCA results for TNSH.The evolutionary tree results revealed a close genetic relationship between TNSH and QR, while KE and CM were situated on the same evolutionary branch (Figure 1A).

### 3.2 Selective sweeps

#### 3.2.1 Fixation index

Initially, we ranked the Fst values of TNSH in descending order and selected the top 5% as selection regions. After Fst filtering, we identified a total of 3048 genes. When examining the degree of differentiation between KE and TNSH populations using Fst, we discovered the gene *PDGFD*, which influences tail fat deposition. Fst tests for QR, CM, and KE revealed 2723,3264 candidate genes, respectively. (Supplementary Data 1).

#### 3.2.2 Haplotype statistics

**FIGURE 2:**
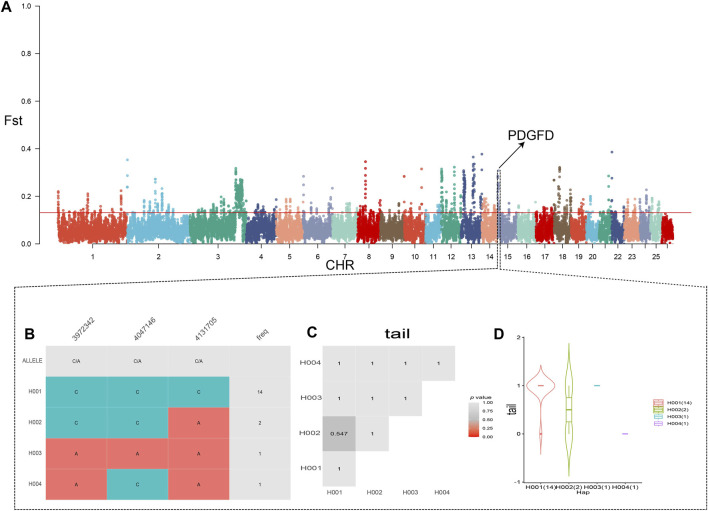
**(A)** Fst results of KE and TNSH, the red line in the figure represents the 0.05 threshold **(B)** Haplotype analysis of the region near *PDGFD* (chr15:3800010-4200000) based on the genehap R package. **(C, D)** Phenotypic correlation analyses of the fat tail of Kirghiz sheep with the thin tail of TNSH).

Subsequently,we generated tail trait phenotype-associated haplotypes in the *PDGFD* gene region,and the results showed that the H001 and H003 haplotypes were positively correlated with tail fat deposition in the *PDGFD* gene (Figure 2, Supplementary Data 2).

#### 3.2.3 Nucleotide diversity

**FIGURE 3:**
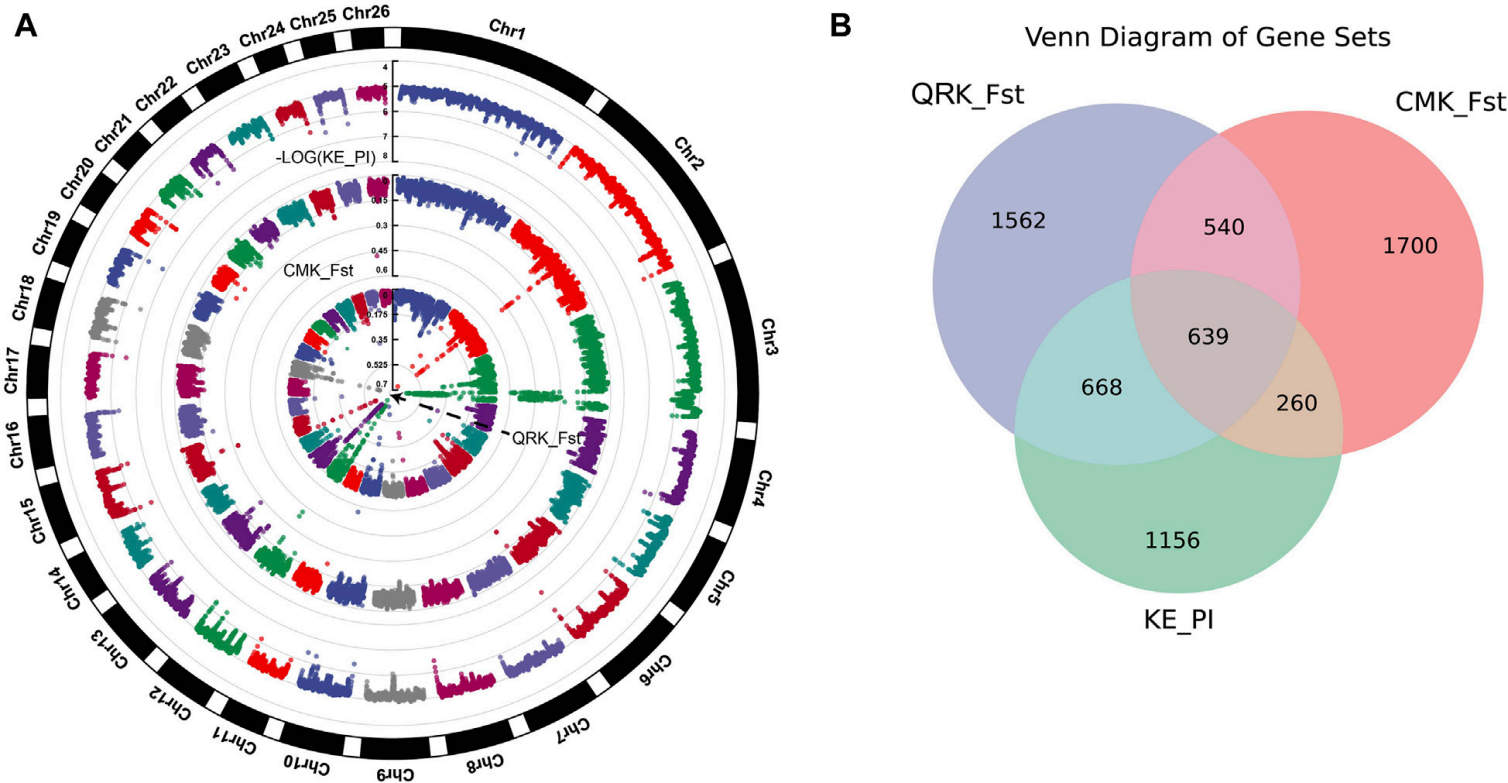
**(A)** Plot of Fst and PI results for KE vs. QR and CM,with different colored dots representing whether the locus is under selection, using a significance threshold of 0.05. **(B)** Intersection of genes annotated from nucleotide diversity in KE and the top 5% Fst loci from KE. QR and CM comparisons).

We sorted the PI values of KE in ascending order and selected the top 5% as the selection region, revealing a total of 3484 genes (Supplementary Data 3). Following the cross-analysis of genes filtered by both Fst and PI, we identified 639 intersecting candidate genes (Figure 3B, Supplementary Data 3).

Through Fst and PI analysis of KE, CM, and QR, we identified several genes associated with Kirghiz sheep. These genes are related to the high-altitude adaptation of organisms, such as *ASIP, FMO1, FMO2, FMO4*, as well as genes related to animal growth and reproduction, such as *PTK6, PAG11*, and *CDC16.*

#### 3.2.4 Candidate gene enrichment analysis

The GO enrichment pathway analysis was performed on 590 candidate genes selected by QR and KE. There were 12 significant *p*-value<0.05 pathways, among which the following were related to high altitude adaptation:GO:0050732∼negative regulation of peptidyl-tyrosine phosphorylation,GO:0006805∼xenobiotic metabolic process, KEGG enrichment pathways showed 18 significant *p*-value<0.05 pathways, among which OAS00430-Taurine and hypotaurine metabolism were associated with high altitude adaptation.oas04610∼Complement and coagulation cascades; oas04672∼Intestinal immune network for IgA production (Supplementary Data 4, Figure 4).

**Table:** 

A:GO enrichment pathway		
Category	Function	Pathway
1	Negative cytoskeleton protein depolymerizatio	regulation of cytoskeleton organization actin cytoskeleton reorganization biological process involved in interaction with host lysosomal transport negative regulation of protein depolymerization
2	Fatty hexose acid oxidative	organic acid metabolic process monosaccharide metabolic process hexose metabolic process
3	ATP diphosphate ADP phhosphorylation	nucleoside diphosphate phosphorylation glycolytic process ATP generation from ADP nucleotide phosphorylation
4	Adaptive activation immune differentiation	lymphocyte activation positive regulation of immune system process
5	Leukocyte lymphocyte apoptotic process	regulation of leukocyte apoptotic process regulation of lymphocyte apoptotic process lymphocyte apoptotic process

B:KEGG enrichment pathway		
category	function	Pathway
1	Nucleotide absorption scytokine	Protein digestion and absorption Taste transduction Complement and coagulation cascades Viral protein interaction with cytokine and cytokine receptor Intestinal immune network for IgA production Fructose and mannose metabolism Insulin resistance Biosynthesis of nucleotide sugars Amino sugar and nucleotide sugar metabolism
2	Giycolysis amino gluconeogenesis acids	Glycolysis/ Gluconeogenesis HIF-1 signaling pathway
3	Drup hypotaurine cytoc P450	Drug metabolism - cytochrome P450 Taurine and hypotaurine metabolism
4	C-type glucagon herpe infection	Kaposi sarcoma-associated herpesvirus infection C-type lectin receptor signaling pathway
5	Aldosterone-regulated expression checkpoint	Neurotrophin signaling pathway PD-L1 expression and PD-1 checkpoint pathway in cancer T cell receptor signaling pathway

**FIGURE 4:**
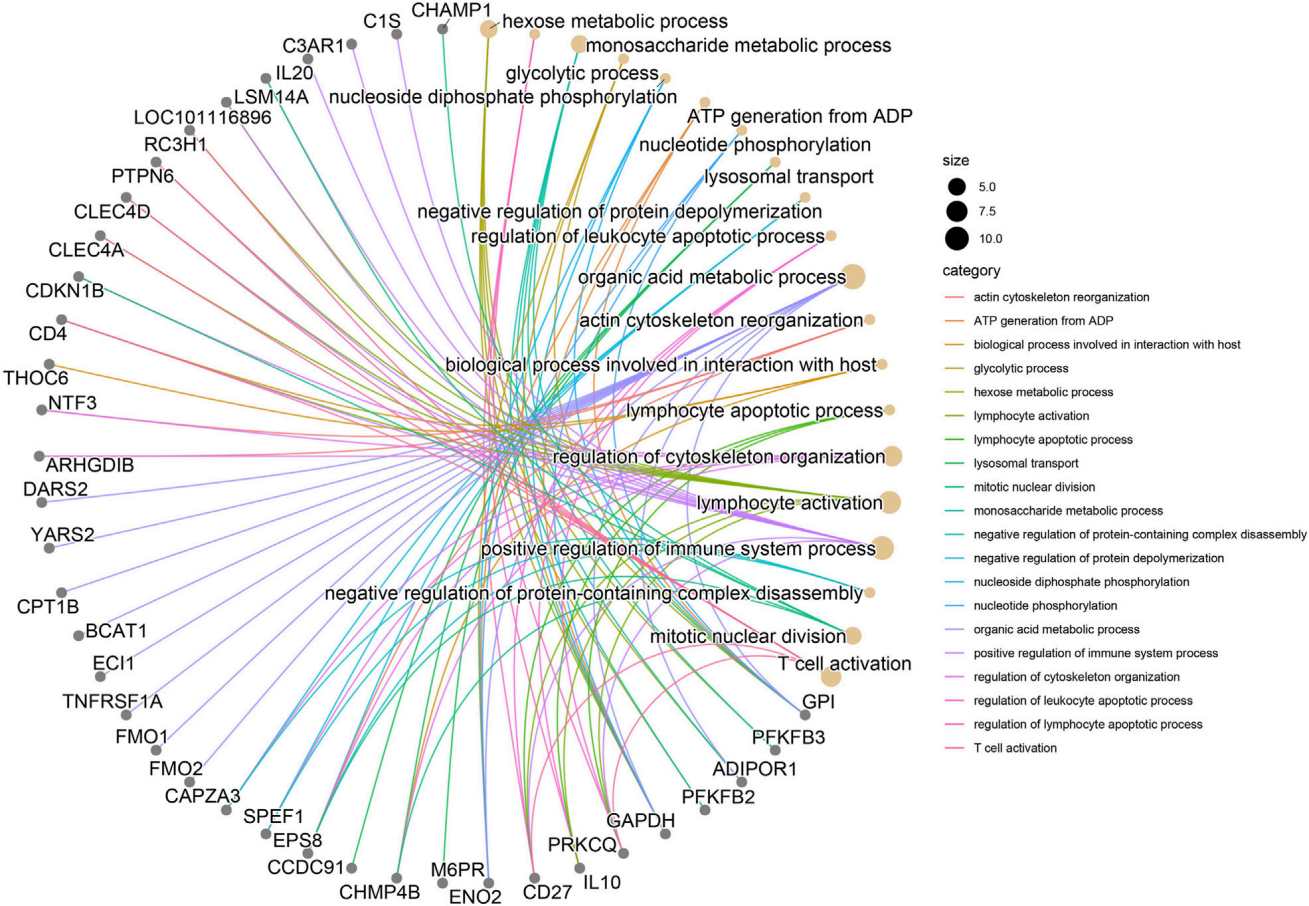
significance threshold of the top twenty key genes for the interaction of enrichment pathway.

## 4 Discussion

### 4.1 Genetic diversity and population structure

Principal component analysis of Kirghiz sheep, Tien-shan sheep, Qira black and Merino sheep showed no significant aggregation in these four populations. However, Qira black and Tien-shan sheep exhibited closer proximity, suggesting a potential shared ancestry, possibly influenced by historical breeding practices.Notably, despite inhabiting the Naryn region of Kyrgyzstan (Deniskova et al., 2019b), Tien-shan sheep demonstrated unstable population structure, likely influenced by human-mediated selection over recent decades (Eydivandi et al., 2021).Conversely, Qira black sheep from Hotan cele County displayed greater ancestral resemblance to Tien-shan sheep, possibly attributable to trade dynamics in livestock (Valerio et al., 2020).

The phylogenetic tree reconstructed analysis indicated no significant kinship among these populations, corroborating the PCA findings and suggesting independent evolutionary trajectories in relatively isolated environments.

However, ancestral proportion analysis revealed a close genetic affinity between Kirghiz and Tien-shan sheep, as well as Qira black sheep. This likely stems from shared ancestral components and genetic distances, potentially shaped by prolonged cohabitation in similar environments and the introduction of foreign breeds, contributing to genetic homogeneity among these populations.

### 4.2 Adaptation mechanism in plateau environment

*ASIP* (agouti signaling protein) located on chromosome 13 of sheep, from physical distance 63063228 to 63063230 bp, *ASIP*, which is an autosomal gene, is directly related to a pathway that regulates melanin production (Goutte et al., 2022).*ASIP* plays a crucial role in regulating changes in the color of the animal’s back skin, It not only affects the muscle tone of the back, but also directly controls the production of melanin in the ventral skin area. It can regulate the process of protein formation in animal skin pigment, by precisely regulating the distribution and activity of melanocytes. Thus, it can be said that *ASIP* has a decisive influence in shaping the color pattern of the animal’s back and the absence or overexpression of melanin in the ventral skin (Li MH. et al., 2014). *ASIP* leads to the synthesis of eumelanin in hair follicle melanocytes, rather than the black or brown pigment true melanin, Gene switching events at *ASIP* sites in sheep may play an important role in the evolution of pigmentation in sheep (Norris and Whan, 2008). The pleiotropic effects expressed in animal models include obesity (Kempf et al., 2022),increased susceptibility to tumors, and premature infertility (Lima et al., 2015). Therefore,we can infer that the formation of *ASIP* is closely related to environmental factors. At higher altitudes, Ultraviolet radiation levels increase significantly, and sheep in these areas may adapt to this change by developing specific physiological mechanisms to reduce Ultraviolet radiation damage to them, which can help sheep better withstand Ultraviolet radiation stress in high-altitude environments. *FMO1* (flavin containing dimethylaniline monoxygenase 1) is an enzyme containing flavin, located on chromosome 12 of sheep, with a physical position between 36894203 and 36894334 bp. Together with P450 enzymes, *FMO1* participates in the reduction of *TNO* (N-oxidized dimethylaniline) to *TAM* (dimethylaniline), and the oxidation of *TAM* to *TNO* (Parte and Kupfer, 2005). FMO is the primary enzyme oxidant in this process,it binds to *NADPH* and exerts its catalytic activity (Eswaramoorthy et al., 2006). *FMO* can regulate cellular stress resistance through various cellular energy metabolism activities such as mitochondrial respiration pathway and glycolysis (Huang et al., 2021). When the levels of mean erythrocyte hemoglobin and mean erythrocyte hemoglobin concentration in the blood are elevated, it may indicate that sheep exhibit enhanced oxygen carrying capacity. *FMO1* as a regulator of energy homeostasis (Veeravalli et al., 2014), is highly expressed in the liver and kidney of rats (Lattard et al., 2002) but can cause dysregulation of lipid metabolism (Zou et al., 2023), suggesting improved adaptation to high altitude hypoxia (Zhang Y. et al., 2021). This helps the Kirghiz adapt to the low oxygen and cold conditions at high altitudes. *FMO2* (flavin containing dimethylaniline monoxygenase 2) gene, located on chromosome 12 of sheep, has a physical position between 36833449 and 36833580 bp.The human *FMO2* gene has been confirmed to regulate oxidative stress levels (Henderson et al., 2008), thus playing a role in innate immunity against microbial infections, including tuberculosis (Mekonnen and Bekele, 2017). the *FMO2* gene as an immune regulatory factor, may play a role in their resistance to tuberculosis. In addition,the transcription of this gene is more than 50% expressed in the lung (Siddens et al., 2008), but not in the liver or kidney (Yueh et al., 1997). The lung pressure, blood oxygen-carrying capacity and lipid metabolism of Kirghiz sheep living in high altitude have important effects on their adaptation to high altitude environment. These results suggest that *FMO1* and *FMO2* genes may play an important role in the cold anoxic environment and immune response mechanism under the plateau, and further studies may reveal their specific functions and mechanisms in Kirghiz sheep population.

### 4.3 Tail fat-related genes

Tail fat plays an important role in the adaptability of the Kirghiz sheep, not only being economically valuable (Zhao et al., 2022), but also storing energy and acting as a buffer to help the sheep survive extremely cold and arid environments (Wang et al., 2021). The deposition of fat in the tail can store energy and act as a buffer, thereby protecting the organism from the effects of extreme environments (Moradi et al., 2022). Additionally, fat is involved in regulating key physiological and biochemical responses in the organism (Zhang W. et al., 2021), crucial for the adaptation of Kirghiz sheep to high-altitude environments.Through selection signal analysis, we discovered candidate genes related to sheep tail fat, located in the CDS region of the PDGFD gene area, from chr15:3800010 to 4200000 bp. Three haplotypes were identified: H001 (chr15:3972342) with a frequency of 14, H002 with a frequency of 2, and H003 (chr15:4131705) with a frequency of 1 (Figure 2). The expression of the PDGFD region is related to the maturation of adipocytes (Luo et al., 2021). Members of the PDGF (prostaglandin-related factors) family have been shown to promote the proliferation of certain types of preadipocytes and effectively inhibit differentiation into mature adipocytes (Ma et al., 2018) (Pan et al., 2019), playing a key role in the process of body fat adipogenesis in humans and mice, and controlling the shape of the fat-tailed sheep to a certain extent (Dong et al., 2020). This gene is important in regulating fat deposition in the thin-tailed sheep (Li et al., 2020). We identified functional genes and selective gene regions associated with fat deposition in Kirghiz sheep breeds, specifically in the PDGFD region.

## 5 Conclusion

In conclusion,we employed the Illumina Ovine SNP50K Bead Chip for targeted signal analysis of Kirghiz sheep thriving in high-altitude environments.Our investigation unveiled candidate genes such as *ASIP, FMO1, FMO2*, implicated in the growth and development of Kirghiz sheep and their adaptation to elevated altitudes, along with *PDGFD*,a pivotal gene governing fat deposition in sheep tails. Our research outcomes are instrumental for pinpointing candidate genes associated with crucial traits across diverse sheep breeds, addressing the challenges posed by global climate change, cultivating novel sheep varieties endowed with robust cold tolerance,and offering insights into the development of new sheep breeds boasting desirable tail characteristics. This study establishes a solid groundwork for breeding superior new sheep germplasm.

## Data Availability

The data presented in the study are deposited in the Figshare repository, accession link: https://doi.org/10.6084/m9.figshare.25744491.v1.
